# Wide-Angle Scanning Phased Array Antenna using High Gain Pattern Reconfigurable Antenna Elements

**DOI:** 10.1038/s41598-019-54120-2

**Published:** 2019-12-05

**Authors:** ByungKuon Ahn, In-June Hwang, Kwang-Seok Kim, Soo-Chang Chae, Jong-Won Yu, Han Lim Lee

**Affiliations:** 1 Korea Advanced Institute of Science and Technology (KAIST), School of Electrical Engineering, Daejeon, 34141 South Korea; 2 Korea Research Institute of Standards and Science (KRISS), Center for Electromagnetic Metrology, Daejeon, 34113 South Korea; 3 Chung-Ang University, School of Electrical and Electronics Engineering, Seoul, 06974 South Korea

**Keywords:** Electrical and electronic engineering, Engineering

## Abstract

This paper presents a wide-angle scanning phased array antenna using high gain pattern reconfigurable antenna (PRA) elements. Using PRA elements is an attractive solution for wide-angle scanning phased array antennas because the scanning range can be divided into several subspaces. To achieve the desired scanning performance, some characteristics of the PRA element such as the number of switching modes, tilt angle, and maximum half-power beamwidth (HPBW) are required. We analyzed the required characteristics of the PRA element according to the target scanning range and element spacing, and presented a PRA element design guideline for phased array antennas. In accordance with the guideline, the scanning range was set as ±70° and a high gain PRA element with three reconfigurable patterns was used to compose an 8x1 array antenna with 0.9 λ_0_ spacing. After analyzing whether the active element patterns meet the guideline, the array antenna was fabricated and measured to demonstrate the scanning performance. The fabricated array can scan its beam from -70° to 70° by dividing the scanning range into three subspaces. It shows that even if the array antenna has large element spacing, the desired scanning performance can be obtained using the elements designed under the guideline.

## Introduction

The phased array antenna has been comprehensively studied because of its advantages such as agile and flexible beam scanning, high tracking accuracy, and widely used in many military and civil applications such as wireless communication systems, wireless power transmission systems, and radar systems^1^. The main purpose of the phased array antenna is to scan a wide range with high array gain using as few antennas as possible. According to the array antenna theory, the array pattern is consists of the product of array factor and element pattern. The array pattern of a linear array antenna can be simply expressed as equation 1, where $f$ is the element pattern, $N$ is the number of antenna elements, ${k}_{0}$ is the wave number, $d$ is the element spacing, and $\Phi$ is the phase difference between elements. In general, the scanning range of phased array antenna means 3 dB-coverage and is limited by the element pattern due to the relation in equation 1.

1 $$Array\ Pattern=Element\ Pattern\times Array\ Factor=f(\theta )\times \mathop{\sum }\limits_{n=1}^{N}{e}^{-j(n-1)({k}_{0}d\sin \theta -\Phi )}$$

**Figure 1:**
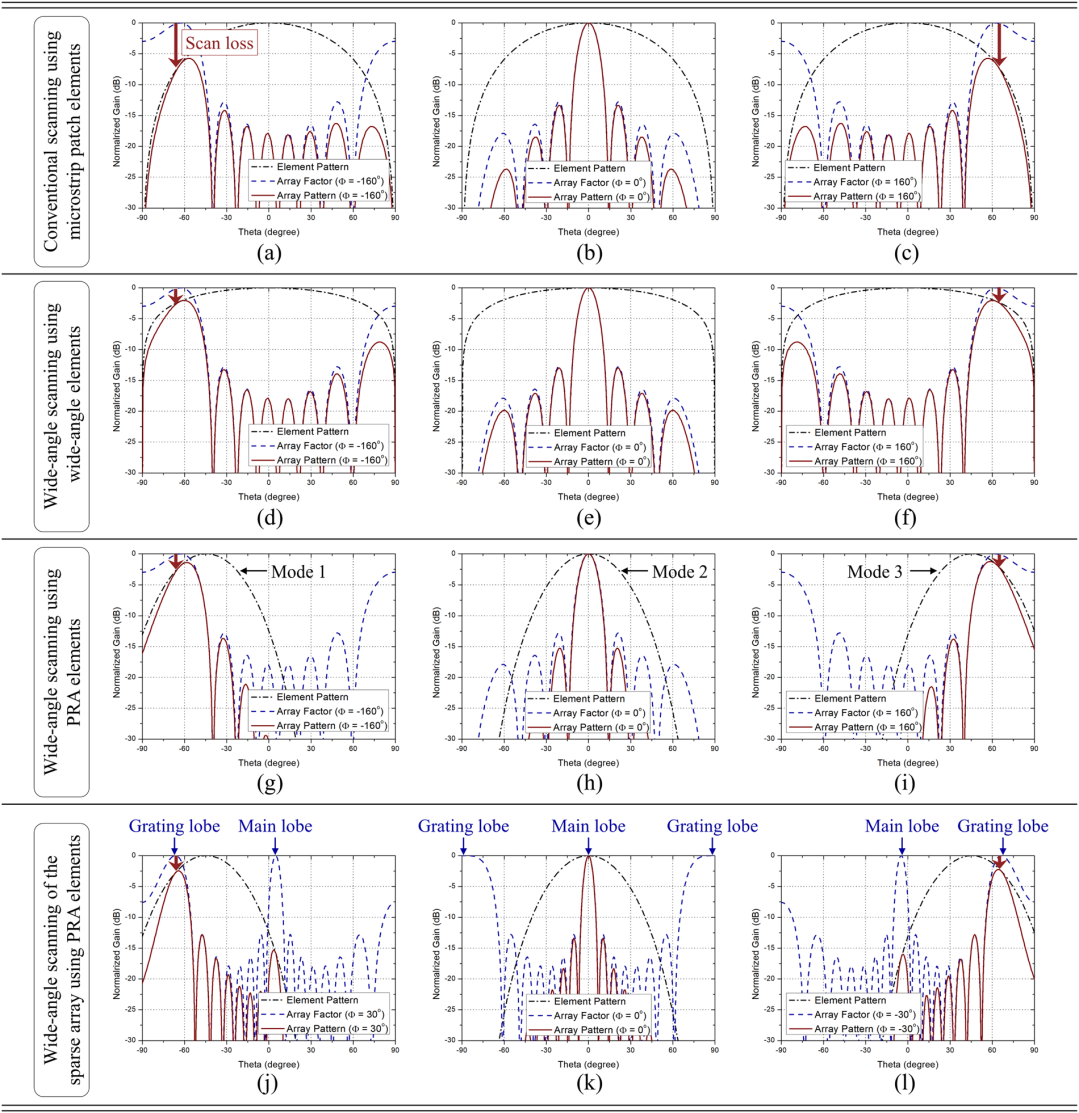
Various beam scanning concepts of the phased array antenna. (**a**–**c**) Conventional scanning using microstrip patch elements. (**d**–**f**) Wide-angle scanning using wide-angle elements. (**g**–**i**) Wide-angle scanning using PRA elements. (**j**–**l**) Wide-angle scanning of the sparse array using PRA elements.

For this reason, the microstrip patch antenna which has a peak gain of about 5dBi and a half-power beamwidth (HPBW) of about ${90}^{\circ }\sim {100}^{\circ }$ is generally used to the array^2^. The normalized array pattern, array factor and element pattern of a phased array antenna using the microstrip patch antenna elements are shown in Fig. 1(a–c). They show the case when the HPBW of antenna element is ${90}^{\circ }$, $N$ is 8, and $d$ is 0.5${\lambda }_{0}$, where ${\lambda }_{0}$ is the free-space wavelength at operating frequency. In Fig. 1(a–c), the phase differences are ${-160}^{\circ }$, ${0}^{\circ }$, and ${160}^{\circ }$, respectively. The black dash-single dotted line, blue dashed line, and red solid line denote the element pattern, array factor, and array pattern, respectively. As the beam is steered to a wider angle, the scan loss increases due to the element pattern and the scanning range is limited. Therefore, in order to achieve the wide-angle scanning essentially, the element pattern should be widened.

Many studies were carried out to realize the wide-angle scanning, and in most studies, the arrays have the scanning range of about ${140}^{\circ }$ or more. The various wide-angle scanning techniques can be categorized into two methods. The first method is using wide-angle elements^3–9^. The wide-angle elements provide a wide scanning range without increasing the complexity of beamforming network. The scanning concepts of a phased array antenna using the wide-angle elements are shown in Fig. 1(d–f). They show the case when the HPBW of antenna element is ${140}^{\circ }$, $N$ is 8, and $d$ is 0.5${\lambda }_{0}$. In Fig. 1(d–f), the phase differences are ${-160}^{\circ }$, ${0}^{\circ }$, and $16{0}^{\circ }$, respectively. It can be seen that since the antenna element has a wide beam pattern, the scan loss is small and a wider range can be scanned. However, the element spacing is limited to 0.5${\lambda }_{0}$ or less to avoid the ambiguity problems caused by grating lobes, so the wide-angle elements should be designed within a physical size smaller than 0.5${\lambda }_{0}$. Also, as the beam is steered to a wider angle, there arises a problem that the side lobe level rises to a non-negligible level.

The second method is using pattern reconfigurable antenna (PRA) elements. The PRA refers to an antenna which can reconfigure its radiation pattern in various directions while maintaining the operating frequency, and it includes a switching network to control the switching modes^10–19^. The scanning concepts of a phased array antenna using the PRA elements are shown in Fig. 1(g–i). They show the case when the antenna element has three reconfigurable patterns whose main beam directions are ${-47}^{\circ }$, ${0}^{\circ }$, ${47}^{\circ }$ and HPBWs are all ${47}^{\circ }$. The switching modes in each status are named as Mode 1, Mode 2, and Mode 3, respectively. As with the previous cases, $N$ is 8, and $d$ is 0.5${\lambda }_{0}$. In Fig. 1(g–i), the phase differences are ${-160}^{\circ }$, ${0}^{\circ }$, and ${160}^{\circ }$, respectively. Since the element pattern is reconfigurable, the scanning range can be divided into several subspaces by controlling the switching mode to generate the element beam in the desired direction. As shown in Fig. 1(g–i), when the switching mode of the antenna element is Mode 1, Mode 2, and Mode 3, the range of Subspace I, Subspace II, and Subspace III is respectively scanned. Another advantage of using PRA elements is that the grating lobes can be utilized as the main beam. The PRA elements often have antenna sizes larger than 0.5${\lambda }_{0}$ due to the addition of the multiple feeding networks, parasitic elements, and switching networks. In these cases, because the element spacing should be set wide, the array becomes a sparse array and the grating lobes occur in the array factor. However, using PRA elements can attenuate the array factor not included in the desired subspace so the ambiguity problems caused by grating lobes can be solved. The scanning concepts of a sparse phased array antenna using the PRA elements are shown in Fig. 1(j–l). They show the case when the characteristics of the PRA element are the same as in Fig. 1(g–i), but $d$ is 1.0${\lambda }_{0}$. In Fig. 1(j–l), the phase differences are $-3{0}^{\circ }$, ${0}^{\circ }$, and ${30}^{\circ }$, respectively. Since it is a sparse array with large element spacing, we can see that the grating lobes occur in the array factor. However, when the element can reconfigure its beam pattern in the direction of the desired subspace, the array factor not included in the range of desired subspace is attenuated so that the main lobe and the grating lobes can be selectively used as the main beam. In other words, the scanning range can be scanned by utilizing the grating lobes, rather than avoiding them. Further, even if the array antenna has large element spacing, there is no problem in beam scanning if the element has a sufficient number of reconfigurable modes. Therefore, it is free from the size limitation of the antenna element. The scanning performances shown in Fig. 1 are the theoretical calculation results assuming that all element patterns are the same. In array antennas, the active element pattern is different from the single element pattern due to the mutual coupling among elements and the structural influence of array, so that some errors occur and the scanning range become wider or narrower than theoretical prediction^20–25^. Therefore, it is important to analyze how the active element pattern changes when the elements are arranged. The accurate beam scanning performance can be predicted by considering the active element pattern. Meanwhile, the number of antenna elements required to obtain the desired array gain depends on the element gain. The higher the element gain, the fewer elements are required. In general, when the element gain increases 3 dB, the number of elements can be reduced by half^1^. Moreover, the use of high gain elements can reduce the number of active devices such as low noise amplifiers (LNAs), phase shifters, and power amplifiers which are used to compose the beamforming network. One of the practical problems in implementing the phased array antenna is the heat problem occurred by the active devices. The use of high gain elements can not only reduce the number of active devices but also mitigate the heat problem. Due to these advantages, high gain antenna elements have been studied steadily, and in most studies, the antennas have peak gains of 9 dBi or more^26–30^. However, when the high gain elements are used to an array, the scanning range is narrow because the element pattern is narrow. Thus, if a phased array antenna is composed of the elements which have high gain and can reconfigure its beam pattern, a superb wide-angle scanning performance with high array gain can be achieved using a small number of antenna elements. To analyze the scanning performance of the phased array antennas composed of PRA elements, there are many factors to consider such as element spacing, number of switching modes, tilt angle in each mode, and HPBW. Analyzing and deriving the characteristics of the PRA elements required to achieve the desired scanning performance of the phased array antenna will be a very useful design guideline for phased array antennas using PRA elements.

In this paper, we present a wide-angle scanning phased array antenna using high gain PRA elements. First of all, we derive the required characteristics of the PRA element such as the number of switching modes, tilt angle in each mode, and maximum HPBW to achieve the desired scanning performance under the condition of element spacing. Based on the derivation, we present a PRA element design guideline for achieving the desired scanning performance of phased array antennas. When the PRA elements designed under the guideline are used, the array antenna can achieve the desired scanning performance by dividing the scanning range into several subspaces. To achieve a scanning range of $$\pm $$$${70}^{\circ }$$ in accordance with the guideline, we used a high gain PRA model proposed by our research group^19,29^. The proposed high gain PRA model can reconfigure its beam pattern in three directions using a reconfigurable switching network and the size is 0.53$${\lambda }_{0}$$. We composed a 8x1 array antenna with 0.9$${\lambda }_{0}$$ spacing and analyzed whether the active element patterns meet the guideline. Afterward, it is experimentally validated that the desired scanning performance of the proposed array antenna can be obtained by dividing the scanning range into three subspaces. The measured peak gain of the prototype array antenna is about 17 dBi, and the measured scanning range is from $${-70}^{\circ }$$ to $${70}^{\circ }$$ with gain fluctuation less than 3 dB. In all scanning range, the side lobe level is less than $$-10$$ dB. Since the proposed array antenna is composed of the high gain PRA elements, wide-angle scanning performance with high array gain can be achieved with a few antenna elements and it is expected to be applicable to various applications of the phased array antenna.

## Results

### PRA element design guideline for phased array antennas

Generally, the element spacing of the phased array antenna is strictly limited to 0.5${\lambda }_{0}$ or less to avoid the ambiguity problems caused by grating lobes. However, for the phased array antennas composed of PRA elements, the element spacing is no longer limited because the grating lobes can be utilized as the main beam. In order to analyze the scanning performance of the phased array antenna composed of PRA elements, the formula of antenna gain, equation 2, is used to simply generate the desired element pattern, where $G$ is the antenna gain, ${e}_{cd}$ is the radiation efficiency, $D$ is the directivity, $U$ is the radiation intensity, and ${P}_{rad}$ is the total radiation power^2^. The element beam patterns with the main beam direction in ${0}^{\circ }$ are generated by setting the radiation intensity $U$ as ${\cos }^{n}\theta$ and varying the value $n$. The radiation efficiency ${e}_{cd}$ is assumed to be 1. The tilted beam patterns by pattern reconfiguration can be obtained by extracting the visible region beam pattern and shifting it to the desired angle.

2 $$G(\theta ,\varphi )={e}_{cd}D(\theta ,\varphi )={e}_{cd}\frac{4\pi U(\theta ,\varphi )}{{P}_{rad}}\,(U(\theta ,\varphi )={\cos }^{n}\theta ,{P}_{rad}={\int }_{0}^{2\pi }{\int }_{0}^{\pi }U(\theta ,\varphi )\sin \theta d\theta d\varphi )$$

The first parameter to be set for analyzing the scanning performance is the target scanning range. The target scanning range is set as $$\pm {\theta }_{max}$$ (total $$2{\theta }_{max}$$). The concept of the beam scanning using PRA elements is to scan each subspace by dividing the scanning range into $$M$$ subspaces, where $$M$$ is the number of switching modes of the PRA element. The subspace range of each switching mode can be expressed as equation 3 by dividing the scanning range by $$M$$ equally. The ideal tilt angle of the PRA element in each switching mode is the center of subspace and can be expressed as equation 4. For instance, in the case that the target scanning range is $$\pm $$$${70}^{\circ }$$ and the PRA element has three switching modes, the three subspaces Subspace I, Subspace II, Subspace III are calculated as $${-70}^{\circ }\sim {-23.3}^{\circ }$$, $${-23.3}^{\circ } \sim 23.{3}^{\circ }$$, $$23.{3}^{\circ } \sim 7{0}^{\circ }$$, respectively, and the ideal tilt angle $${\theta }_{1}$$, $${\theta }_{2}$$, $${\theta }_{3}$$ are calculated as $${-46.7}^{\circ }$$, $${0}^{\circ }$$, $${46.7}^{\circ }$$, respectively.

3 $$Subspace\ K\ :\ \frac{2(K-1)-M}{M}{\theta }_{max}\le \theta \le \frac{2K-M}{M}{\theta }_{max}\quad (K=1,2,3,\cdots \ ,M)$$

4 $$Tilt\ angle,\ {\theta }_{k}=\left(\frac{2k-M-1}{M}\right){\theta }_{max}\quad (k=1,2,3,\cdots \ ,M)$$

After setting the subspaces and tilt angle, the maximum HPBW of the antenna element ($HPB{W}_{max}$) should be set, not to invade the undesired subspaces. Otherwise, the ambiguity problems occur due to the grating lobes. For determining $HPB{W}_{max}$, the minimum angular difference between the main lobe and grating lobes is used as a criterion. The dominant parameter generating the main lobe and grating lobes in the array factor is the element spacing $d$. In the array factor formula in equation 1, the main lobe and grating lobes occur in the angle $\theta$ which satisfying the equation 5.

5 $${k}_{0}d\sin \theta -\Phi =2m\pi \,(m=\pm \,0,1,2,3,\cdots )$$

6 $$Main\ lobe\ angle,\ {\theta }_{ML}={\sin }^{-1}\left(\frac{\Phi }{{k}_{0}d}\right)$$

7 $$Grating\ lobes\ angle,\ {\theta }_{GL}={\sin }^{-1}\left(\frac{2m\pi +\Phi }{{k}_{0}d}\right)\quad (m=\pm \,1,2,3,\cdots \ )$$

The main lobe occurs in the angle ${\theta }_{ML}$ when $m=0$, and the grating lobes occur in the angle ${\theta }_{GL}$ when $$m=\pm 1,2,3,\cdots \ $$. The minimum angular difference between the main lobe and grating lobe (${\theta }_{gap}$) always occurs between two lobes generated symmetrically relative to ${0}^{\circ }$ when the phase difference $\Phi$ is ${180}^{\circ }$ and can be expressed as equation 8.

8 $$\ \ {\theta }_{gap}=min| {\theta }_{ML}-{\theta }_{GL}| =2\ {\sin }^{-1}\left(\frac{\pi }{{k}_{0}d}\right)$$

The value of ${\theta }_{gap}$ is used as the criterion for determining $HPB{W}_{max}$. If $HPB{W}_{max}$ is set equal to ${\theta }_{gap}$ value, the ambiguity problems still exist because the two lobes can be contained in one subspace. Thus, a slight margin is required to switch the mode before ambiguity problems occur. By iteration, we determined that when the element’s $-5$ dB beamwidth is equal to ${\theta }_{gap}$, the HPBW of the element is the appropriate $HPB{W}_{max}$. The $-5$ dB beamwidth is the angle between two directions in which the gain is $-5$ dB lower than peak gain. When $HPB{W}_{max}$ is determined, the minimum number of switching modes can be derived. As shown in equation 9, the minimum number of $M$ can be derived based on the value obtained by dividing the scanning range $2{\theta }_{max}$ by $HPB{W}_{max}$. For instance, when the target scanning is $\pm 7{0}^{\circ }$ and the element spacing is 0.9${\lambda }_{0}$, $HPB{W}_{max}$ is calculated as 54.4${}^{\circ }$ and it divides the scanning range by 2.6. Hence, the antenna element should have at least three switching modes and according to equation 4, the tilt angle in each mode should be $-46.{7}^{\circ }$, ${0}^{\circ }$, and ${46.7}^{\circ }$, respectively. In other words, according to the target scanning range and element spacing, the required characteristics of an ideal PRA element such as the minimum number of $M$, tilt angle ${\theta }_{k}$, and $HPB{W}_{max}$ can be derived.

9 $$The\ number\ of\ switching\ modes\ :\ M\ (M\ge 2,\ integer)\ when\ M-1 < \frac{2{\theta }_{max}}{HPB{W}_{max}}\le M$$

By expanding the discussion, the required characteristics of an ideal PRA element can be generalized according to the target scanning range and element spacing, and a PRA element design guideline for phased array antennas can be presented. Representatively, the case where the scanning range is $\pm 7{0}^{\circ }$ is described in detail. When the scanning range is $\pm 7{0}^{\circ }$, the required characteristics of the PRA element according to the element spacing can be calculated as shown in Table 1. When the element spacing is 0.6${\lambda }_{0}$ or 0.7${\lambda }_{0}$, the minimum number of $M$ is 2 because $1 < (2{\theta }_{max}/HPB{W}_{max})\le 2$, and the tilt angle in each mode should be ${-35}^{\circ }$ and ${35}^{\circ }$, respectively. When the element spacing is 0.8${\lambda }_{0}$ or 0.9${\lambda }_{0}$ or 1.0${\lambda }_{0}$, the minimum number of $M$ is 3 because $2 < (2{\theta }_{max}/HPB{W}_{max})\le 3$, and the tilt angle in each mode should be ${-46.7}^{\circ }$, ${0}^{\circ }$, and ${46.7}^{\circ }$, respectively. When the element spacing is 1.1${\lambda }_{0}$ or 1.2${\lambda }_{0}$, the minimum number of $M$ is 4 because $3 < (2{\theta }_{max}/HPB{W}_{max})\le 4$, and the tilt angle in each mode should be ${-52.5}^{\circ }$, ${-17.5}^{\circ }$, ${17.5}^{\circ }$, and ${52.5}^{\circ }$, respectively. When the element spacing is 1.3${\lambda }_{0}$ or 1.4${\lambda }_{0}$ or 1.5${\lambda }_{0}$, the minimum number of $M$ is 5 because $4 < (2{\theta }_{max}/HPB{W}_{max})\le 5$, and the tilt angle in each mode should be ${-56}^{\circ }$, ${-28}^{\circ }$, ${0}^{\circ }$, ${28}^{\circ }$, and ${56}^{\circ }$, respectively.

**Table 1:** The PRA element design guideline for phased array antennas when the target scanning range is $\pm 7{0}^{\circ }$.

$d$	${\theta }_{gap}$	$HPB{W}_{max}$	$M$	$Tilt\ angle$	$Subspace$
0.5${\lambda }_{0}$	180${}^{\circ }$	180${}^{\circ }$	$\ge$1	x	x
0.6${\lambda }_{0}$	112.9${}^{\circ }$	91.0${}^{\circ }$	$\ge$2	${\theta }_{1}=(-1/2){\theta }_{max}=-3{5}^{\circ }$	$Subspace\ I:(-2/2){\theta }_{max} \sim (0/2){\theta }_{max}=-7{0}^{\circ } \sim {0}^{\circ }$
0.7${\lambda }_{0}$	91.2${}^{\circ }$	72.4${}^{\circ }$		${\theta }_{2}=(1/2){\theta }_{max}=3{5}^{\circ }$	$Subspace\ II:(0/2){\theta }_{max} \sim (2/2){\theta }_{max}={0}^{\circ } \sim 7{0}^{\circ }$
0.8${\lambda }_{0}$	77.4${}^{\circ }$	61.0${}^{\circ }$	$\ge$3	${\theta }_{1}=(-2/3){\theta }_{max}=-46.{7}^{\circ }$	$Subspace\ I:(-3/3){\theta }_{max} \sim (-1/3){\theta }_{max}=-7{0}^{\circ } \sim -23.{3}^{\circ }$
0.9${\lambda }_{0}$	67.5${}^{\circ }$	54.2${}^{\circ }$		${\theta }_{2}=(0/3){\theta }_{max}={0}^{\circ }$	$Subspace\ II:(-1/3){\theta }_{max} \sim (1/3){\theta }_{max}=-23.{3}^{\circ } \sim 23.{3}^{\circ }$
1.0${\lambda }_{0}$	60.0${}^{\circ }$	47.6${}^{\circ }$		${\theta }_{3}=(2/3){\theta }_{max}=46.{7}^{\circ }$	$Subspace\ III:(1/3){\theta }_{max} \sim (3/3){\theta }_{max}=23.{3}^{\circ } \sim 7{0}^{\circ }$
1.1${\lambda }_{0}$	54.1${}^{\circ }$	42.6${}^{\circ }$	$\ge$4	${\theta }_{1}=(-3/4){\theta }_{max}=-52.{5}^{\circ }$	$Subspace\ I:(-4/4){\theta }_{max} \sim (-2/4){\theta }_{max}=-7{0}^{\circ } \sim -3{5}^{\circ }$
1.2${\lambda }_{0}$	49.2${}^{\circ }$	38.2${}^{\circ }$		${\theta }_{2}=(-1/4){\theta }_{max}=-17.{5}^{\circ }$	$Subspace\ II:(-2/4){\theta }_{max} \sim (0/4){\theta }_{max}=-3{5}^{\circ } \sim {0}^{\circ }$
				${\theta }_{3}=(1/4){\theta }_{max}=17.{5}^{\circ }$	$Subspace\ III:(0/4){\theta }_{max} \sim (2/4){\theta }_{max}={0}^{\circ } \sim 3{5}^{\circ }$
				${\theta }_{4}=(3/4){\theta }_{max}=52.{5}^{\circ }$	$Subspace\ IV:(2/4){\theta }_{max} \sim (4/4){\theta }_{max}=3{5}^{\circ } \sim 7{0}^{\circ }$
1.3${\lambda }_{0}$	45.2${}^{\circ }$	34.8${}^{\circ }$	$\ge$5	${\theta }_{1}=(-4/5){\theta }_{max}=-5{6}^{\circ }$	$Subspace\ I:(-5/5){\theta }_{max} \sim (-3/5){\theta }_{max}=-7{0}^{\circ } \sim -4{2}^{\circ }$
1.4${\lambda }_{0}$	41.8${}^{\circ }$	32.3${}^{\circ }$		${\theta }_{2}=(-2/5){\theta }_{max}=-2{8}^{\circ }$	$Subspace\ II:(-3/5){\theta }_{max} \sim (-1/5){\theta }_{max}=-4{2}^{\circ } \sim -1{4}^{\circ }$
1.5${\lambda }_{0}$	38.9${}^{\circ }$	29.8${}^{\circ }$		${\theta }_{3}=(0/5){\theta }_{max}={0}^{\circ }$	$Subspace\ III:(-1/5){\theta }_{max} \sim (1/5){\theta }_{max}=-1{4}^{\circ } \sim 1{4}^{\circ }$
				${\theta }_{4}=(2/5){\theta }_{max}=2{8}^{\circ }$	$Subspace\ IV:(1/5){\theta }_{max} \sim (3/5){\theta }_{max}=1{4}^{\circ } \sim 4{2}^{\circ }$
				${\theta }_{5}=(4/5){\theta }_{max}=5{6}^{\circ }$	$Subspace\ V:(3/5){\theta }_{max} \sim (5/5){\theta }_{max}=4{2}^{\circ } \sim 7{0}^{\circ }$

**Figure 2:**
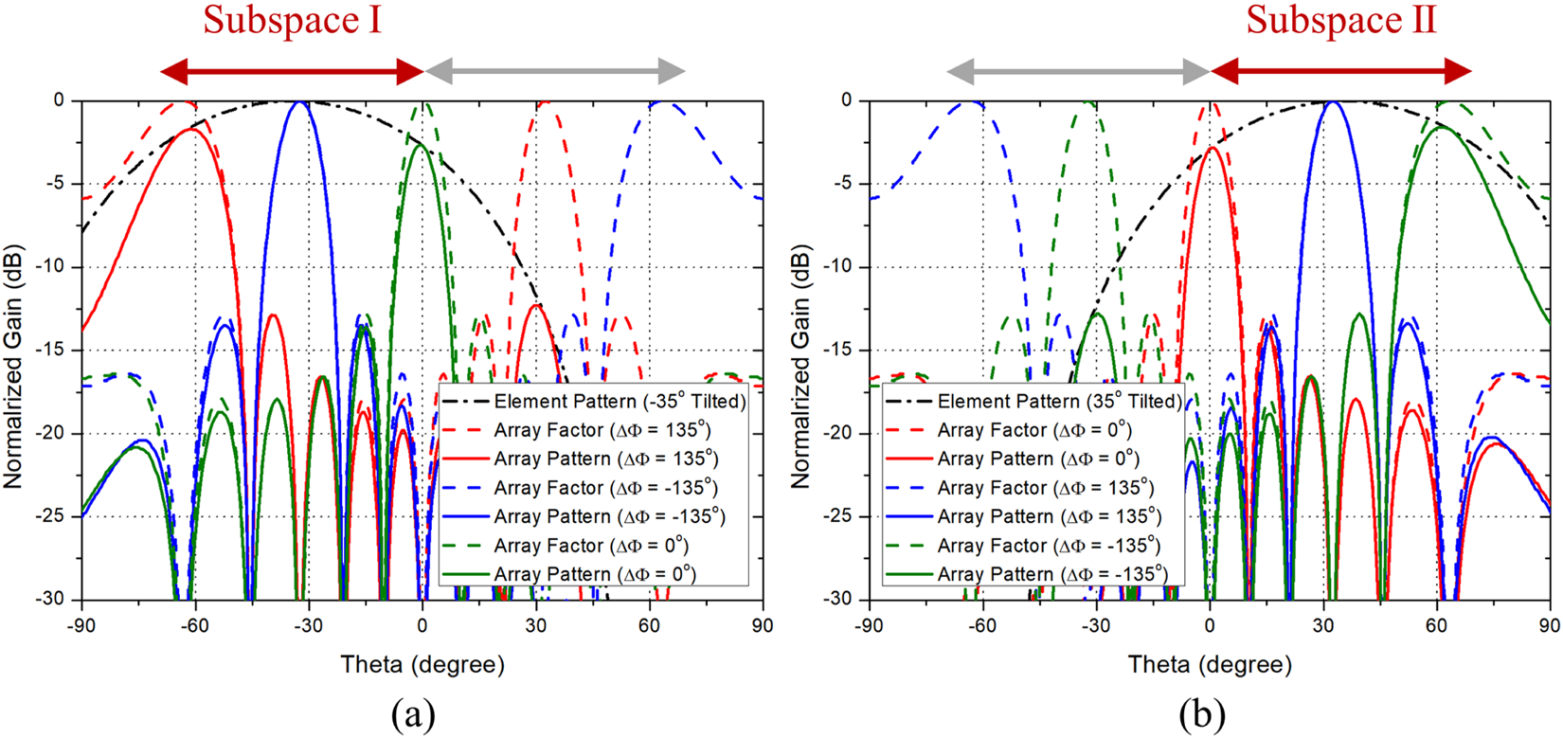
Scanning performance of the case when when $M$ = 2, $N$ = 8, ${\theta }_{max}=7{0}^{\circ }$, and $d$ = 0.7${\lambda }_{0}$. (**a**) Mode 1 (${\theta }_{1}={-35}^{\circ }$). (**b**) Mode 2 (${\theta }_{2}=3{5}^{\circ }$).

**Figure 3:**
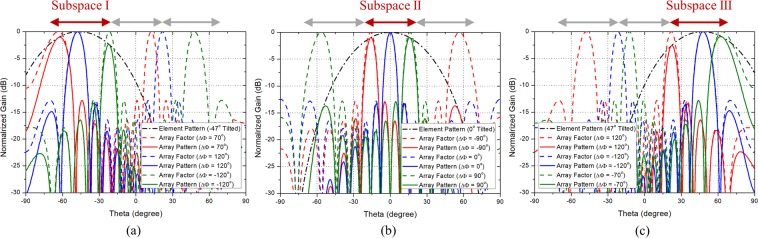
Scanning performance of the case when $M$ = 3, $N$ = 8, ${\theta }_{max}=7{0}^{\circ }$, and $d$ = 0.9${\lambda }_{0}$. (**a**) Mode 1 (${\theta }_{1}={-46.7}^{\circ }$). (**b**) Mode 2 (${\theta }_{2}={0}^{\circ }$). (**c**) Mode 3 (${\theta }_{3}={46.7}^{\circ }$).

**Figure 4:**
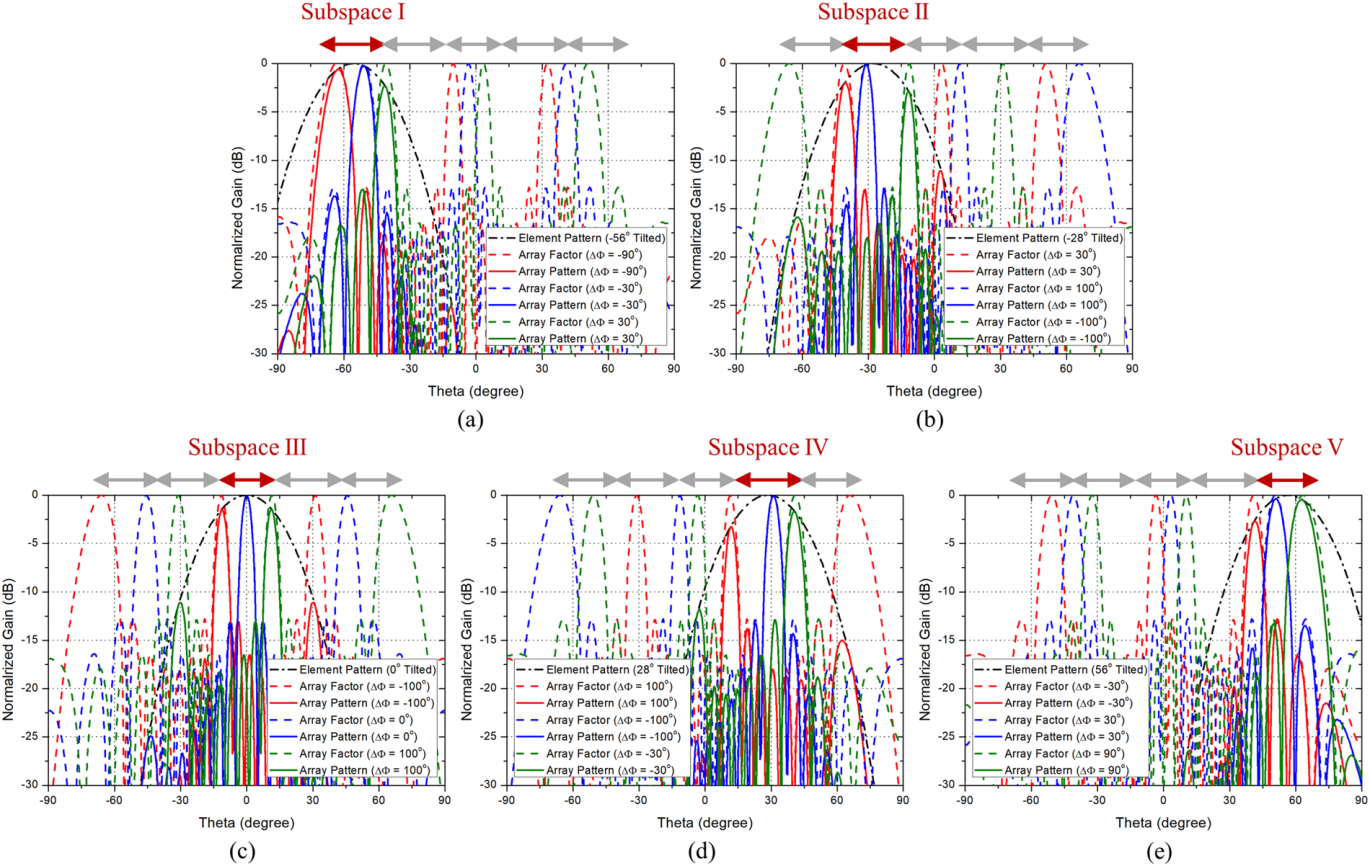
Scanning performance of the case when $M$ = 5, $N$ = 8, ${\theta }_{max}=7{0}^{\circ }$, and $d$ = 1.5${\lambda }_{0}$. (**a**) Mode 1 (${\theta }_{1}={-56}^{\circ }$). (**b**) Mode 2 (${\theta }_{2}={-28}^{\circ }$). (**c**) Mode 3 (${\theta }_{3}={0}^{\circ }$). (**d**) Mode 4 (${\theta }_{4}=2{8}^{\circ }$). (**e**) Mode 5 (${\theta }_{5}=5{6}^{\circ }$).

To show the scanning performance of the phased array antennas composed of the PRA elements which follow the guideline, the scanning patterns when the PRA element has two, three, and five switching modes are shown in Figs. 2, 3, and 4, respectively. Figure 2 shows the scanning performance of the case when $N$ is 8, ${\theta }_{max}$ is ${70}^{\circ }$, and $d$ is 0.7${\lambda }_{0}$. In accordance with the guideline, the PRA element has two switching modes with tilt angle of ${-35}^{\circ }$ and ${35}^{\circ }$. Figure 2(a,b) show the scanning performance of Mode 1 and Mode 2, respectively. From equation 6, the main lobe occurs in ${-45.6}^{\circ }\sim 45.{6}^{\circ }$ depending on the phase difference. So as shown in Fig. 2(a), in Mode 1, the range of ${-45.6}^{\circ } \sim {0}^{\circ }$ can be scanned using the main lobe, and the range of ${-70}^{\circ }\sim {-45.6}^{\circ }$ can be scanned using the grating lobes. Also shown in Fig. 2(b), in Mode 2, the range of ${0}^{\circ } \sim 45.{6}^{\circ }$ can be scanned using the main lobe, and the range of $45.{6}^{\circ } \sim 7{0}^{\circ }$ can be scanned using the grating lobes. It can be seen that the grating lobes can be utilized as the main beam. Figure 3 shows the scanning performance of the case when $N$ is 8, ${\theta }_{max}$ is ${70}^{\circ }$, and $d$ is 0.9${\lambda }_{0}$. In accordance with the guideline, the PRA element has three switching modes with tilt angle of ${-46.7}^{\circ }$, ${0}^{\circ }$, and ${46.7}^{\circ }$. Figure 3(a–c) show the scanning performance of Mode 1, Mode 2, and Mode 3, respectively. The scanning performance can be achieved by dividing the scanning range into three subspaces and using the main lobe and grating lobes occurring in each subspace. The following is the case when the element spacing is quite large. Figure 4 shows the scanning performance of the case when $N$ is 8, ${\theta }_{max}$ is ${70}^{\circ }$, and $d$ is 1.5${\lambda }_{0}$. In accordance with the guideline, the PRA element has five switching modes with tilt angle of ${56}^{\circ }$, ${-28}^{\circ }$ and ${0}^{\circ }$. Figure 4(a–e) show the scanning performance of Mode 1, Mode 2, Mode 3, Mode 4, and Mode 5, respectively. In the same way, the scanning performance can be achieved by dividing the scanning range into five subspaces and using the main lobe and grating lobes occurring in each subspace. We can know that if the characteristics of a PRA element such as the number of switching mode, tilt angle, and HPBW meet the guideline, the desired scanning performance can be achieved even if the element spacing is quite large.

What presented in the guideline is the ideal PRA element pattern, so it may be difficult to accurately implement the tilt angle and HPBW in each mode. However, designing a PRA element based on the guideline can be a helpful manual for obtaining the desired scanning performance of the phased array antenna. When the active element patterns of the PRA element meet the guideline, the desired scanning performance can be achieved by dividing the scanning range into several subspaces.

### High gain PRA element with three-directional reconfigurable patterns

To demonstrate the scanning performance of a phased array antenna composed of the PRA elements based on the guideline, we use a three-directional pattern reconfigurable high gain antenna model proposed by our research group^19,29^. The concept of wide-angle scanning phased array antenna using three-directional PRA elements was presented in before^11^. A millmeter-wave wide-angle scanning phased array antenna using a series-fed aperture-coupled microstrip antenna element with three linearly-arranged patches was designed and implemented. In this paper, we implement a wide-angle scanning phased array using a three-directional pattern reconfigurable dielectric resonator antenna (DRA). The simple operation principle of the high gain PRA model is as follows. A dielectric sphere excited by a microstrip patch works as a spherical dielectric resonator antenna (SDRA) operating on $T{E}_{n01}$ mode. On the higher-order resonant mode above $T{E}_{301}$, this SDRA exhibits high gain characteristic and generates a beam in the direction opposite to the position of microstrip patch. As the resonant frequencies of the microstrip patch and the dielectric sphere are well matched, the input impedance and high gain characteristic show good properties. It means that since the resonant frequency of the dielectric sphere is fixed by its structure, the impedance matching method for the microstrip patch can be applied to this SDRA, thereby the size of the microstrip patch can be miniaturized^29^. Moreover, since the spherical dielectric is symmetric and the magnetic field distribution of the resonant mode is symmetric, as the microstrip patch shifts, the magnetic field distribution rotates and the beam tilting phenomenon occurs. The more the microstrip patch shifted from the center, the beam tilted more. Using this beam tilting characteristic, a high gain PRA can be designed that generates the beams in two directions with two microstrip patches. Feeding both patches at the same time can generate a beam in the middle direction, which is the vector sum direction of two beams. We could obtain a high gain PRA by designing a reconfigurable switching network for selecting each patch alone or selecting them simultaneously. The details about the so-obtained process can be found in refs. ^19,29^.

**Figure 5:**
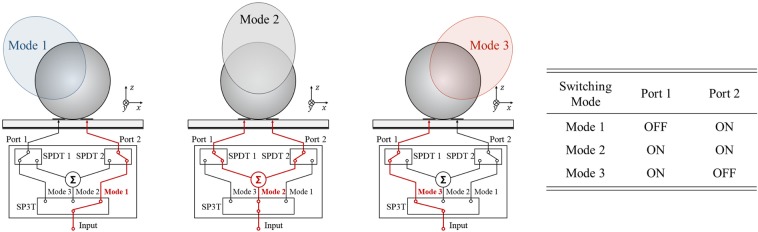
Switching mode configuration of the proposed high gain PRA element.

**Figure 6:**
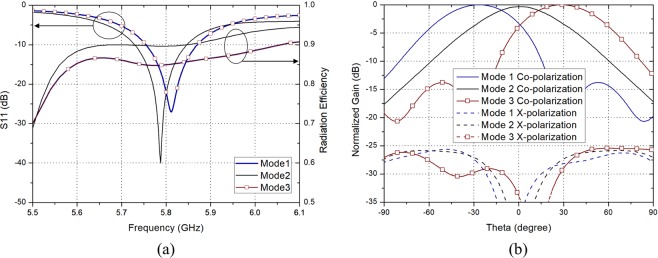
Characteristics of the proposed high gain PRA element. (**a**) Reflection coefficient and radiation efficiency of each mode. (**b**) Radiation pattern of each mode in xz-plane at 5.8 GHz.

 Figure 5 shows the switching configuration of the proposed high gain PRA element. In Mode 1, only the Port 2 is ON and a beam tilted in the negative theta direction is generated. In contrast, In Mode 3, only the Port 1 is ON and a beam tilted in the positive theta direction is generated. In Mode 2, both Port 1 and Port 2 are ON and a beam toward ${0}^{\circ }$ direction which is the vector sum direction of two beams generated by each port is generated. The characteristics of the proposed high gain PRA element are shown in Fig. 6. Figure 6(a,b) show the reflection coefficient of each mode and radiation pattern of each mode in xz-plane at 5.8 GHz, respectively. It is obvious that the proposed antenna element can generate three radiation patterns in different directions. In Mode 1, Mode 2, Mode 3, the peak gains are 9.12 dBi, 8.78 dBi, 9.12 dBi, and the main beam directions are ${-28}^{\circ }$, ${0}^{\circ }$, ${28}^{\circ }$, with the HPBWs of ${52}^{\circ }$, ${54}^{\circ }$, ${52}^{\circ }$, respectively. The radiation efficiency of the proposed antenna shows more than 85% in the operating bandwidth of all switching modes.

**Figure 7:**
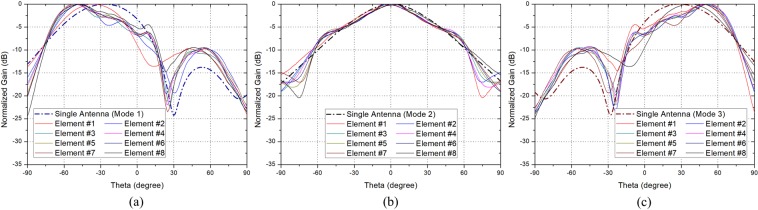
Active element patterns in xz-plane at 5.8 GHz, when eight elements are arranged linearly. (**a**) Mode 1. (**b**) Mode 2. (**c**) Mode 3.

**Figure 8:**
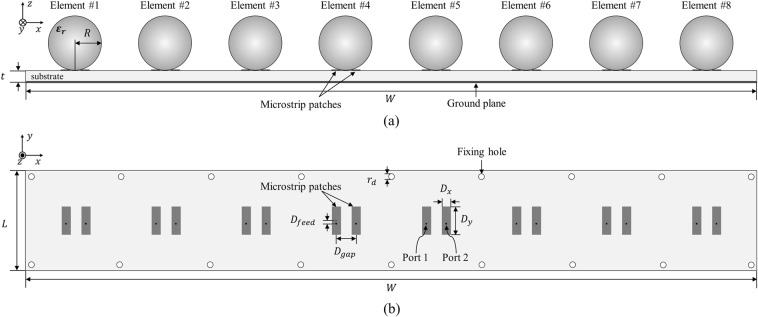
Geometry of the proposed array antenna. (**a**) Cross-sectional view. Parameter: ${\varepsilon }_{r}$ = 13, $R$ = 13.8 mm, $W$ = 365 mm, $t$ = 0.76 mm. (**b**) Top view of the microstrip patches. Parameter: $L$ = 50 mm, ${D}_{x}$ = 4 mm, ${D}_{y}$ = 14 mm, ${D}_{feed}$ = 1.5 mm, ${D}_{gap}$ = 8 mm, ${r}_{d}$ = 3 mm.

Since the antenna size is 0.53${\lambda }_{0}$ and it has three switching modes, it should be arranged with the element spacing of $0.8{\lambda }_{0} \sim 1.0{\lambda }_{0}$ according to the guideline. Considering the value of HPBWs, it seems appropriate to set the element spacing as 0.9${\lambda }_{0}$. Under the same condition of Fig. 3, we analyzed whether the active element patterns are suitable for achieving the scanning range $\pm 7{0}^{\circ }$ when eight elements are arranged with 0.9${\lambda }_{0}$ spacing. Figure 7 shows the comparison between the single antenna radiation pattern and the active element patterns of the array antenna when eight elements are linearly arranged. The elements are named as $Element\ \#1 \sim Element\ \#8$ as shown in Fig. 8. Figure 7(a) shows that the main beam directions of the active element patterns in Mode 1 are more tilted to the negative theta direction because of the mutual coupling among elements and structural influence of array. In the active element patterns in Mode 1, the main beam directions are between ${-38}^{\circ }$ and ${-52}^{\circ }$, and the HPBWs are between $4{5}^{\circ }$ and $5{3}^{\circ }$. The average value of the main beam direction is ${-47.8}^{\circ }$ and the average value of HPBW is $50.{1}^{\circ }$. Contrary, Fig. 7(c) shows that the main beam directions of the active element patterns in Mode 3 are more tilted to the positive theta direction. In the active element patterns in Mode 3, the main beam directions are between ${38}^{\circ }$ and ${53}^{\circ }$, and the HPBWs are between ${43}^{\circ }$ and ${53}^{\circ }$. The average value of the main beam direction is ${48.4}^{\circ }$ and the average value of HPBW is ${50.2}^{\circ }$. Figure 7(b) shows that the active element patterns in Mode 2 are formed symmetrically relative to ${0}^{\circ }$. In the active element patterns in Mode 2, the main beam directions are between ${-7}^{\circ }$ and ${7}^{\circ }$, and HPBWs are between ${52}^{\circ }$ and ${57}^{\circ }$. The average value of the main beam direction is ${0}^{\circ }$ and the average value of HPBW is ${54.1}^{\circ }$. In summary, when the proposed high gain PRA elements are arranged with 0.9${\lambda }_{0}$ spacing, the tilt angle in each mode are ${-47.8}^{\circ }$, ${0}^{\circ }$, ${48.4}^{\circ }$, respectively, and the HPBW in each mode are ${50.1}^{\circ }$, ${54.1}^{\circ }$, ${50.2}^{\circ }$, respectively. Because the characteristics of the proposed antenna element meet the guideline, the element is suitable to array for achieving the desired scanning range of $\pm {70}^{\circ }$. In the next section, we propose an 8x1 phased array antenna using this antenna element, and experimentally validate the wide-angle scanning performance.

**Figure 9:**
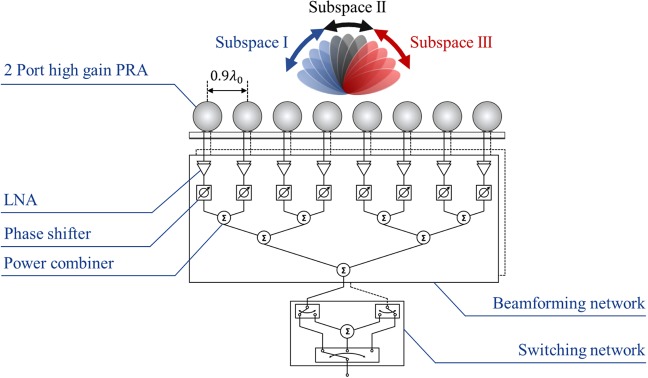
Beamforming system configuration of the proposed array antenna.

**Figure 10:**
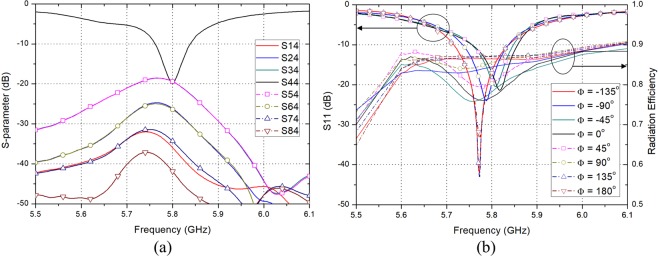
Active S-parameter of the proposed array antenna. (**a**) Simulated S-parameter when $Element\ \#4$ is excited. (**b**) Active reflection coefficient and radiation efficiency of $Element\ \#4$ corresponding to the phase difference.

**Figure 11:**
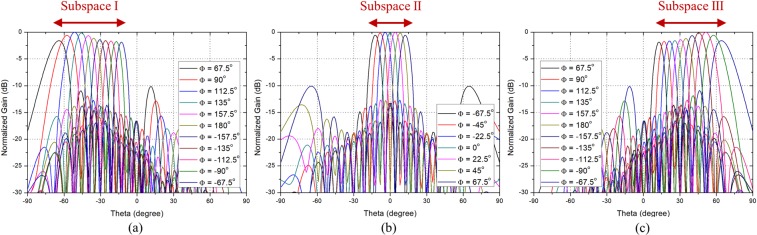
Simulated scanning patterns of the proposed array antenna in xz-plane at 5.8 GHz. (**a**) Mode 1. (**b**) Mode 2. (**c**) Mode 3.

**Figure 12:**
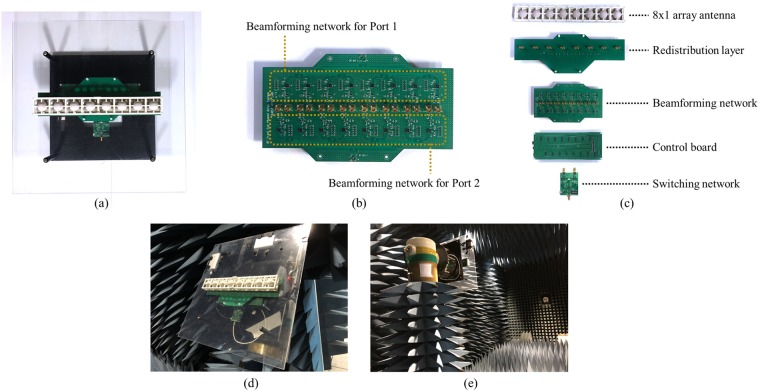
Photographs of prototype array antenna. (**a**) The whole structure including jig. (**b**) Beamforming network. (**c**) Beamforming system configuration. (**d**) Assembled beamforming system.(**e**) Measurement environment.

**Figure 13:**
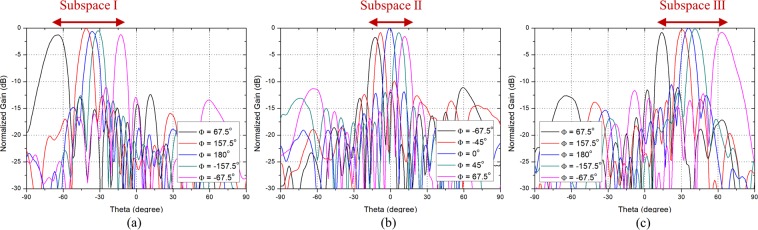
Measured scanning patterns of the prototype array antenna in xz-plane at 5.8 GHz. (**a**) Mode 1. (**b**) Mode 2. (**c**) Mode 3.

### Wide-angle scanning phased array antenna using high gain PRA elements

Since the proposed high gain PRA element was determined to be suitable for scanning $\pm {70}^{\circ }$, we composed an 8x1 phased array antenna using it. The geometry of the proposed array antenna is shown in Fig. 8. Figure 8(a,b) show the cross-sectional view and top view of the proposed array antenna, respectively. Eight ceramic spheres, which dielectric constant is 13, were arranged with 0.9${\lambda }_{0}$ spacing and two microstrip patches were used to excite each sphere. The microstrip patches were printed on a 0.76 mm thickness substrate Taconic RF-301 and fed by Port 1 and Port 2, respectively. For fabrication, there are some holes for fixing the jig structure to hold the dielectric sphere above the microstrip patches. The parameter values on the figure are shown in the caption.

Next, we designed a beamforming network composed of LNA, phase shifter, and combiner to demonstrate the scanning performance of the proposed array antenna. Guerrilla RF GRF2501 and MACOM MAPS-010145 were adopted as LNA and 4bit digital phase shifter, respectively, and Wilkinson power combiner was designed. For pattern reconfiguration of each antenna element, the overall beamforming system is composed of each beamforming network for Port 1 and Port 2 and a reconfigurable switching network combining them. The beamforming system configuration of the proposed array antenna is shown in Fig. 9. As shown in Fig. 5, when the switching mode is Mode 1, only Port 2 of each antenna element is fed so that all elements generate the tilted beam in the negative theta direction and the Subspace I is scanned. Contrary, when the switching mode is Mode 3, only Port 1 of each antenna element is fed so that all elements generate the tilted beam in the positive theta direction and the Subspace III is scanned. When the switching mode is Mode 2, both Port 1 and Port 2 of each antenna element are fed so that all elements generate the beam in the ${0}^{\circ }$ direction and the Subspace II is scanned.

Some of the simulated S-parameters of the proposed array antenna are shown in Fig. 10. Representatively, Fig. 10(a) shows the S-parameter of $Element\ \#4$ in Mode 1. The insertion losses among any elements are more than 18 dB, which demonstrates a weak coupling. Similar results were obtained among any elements or any modes. In Mode 1, the active reflection coefficients of the central element $Element\ \#4$ are shown in Fig. 10(b) when the phase difference between the antenna elements varies from ${-180}^{\circ }$ to ${180}^{\circ }$. As the beam is scanned, the amount of coupling between the elements changes so that the active reflection coefficient curve moves slightly. The active reflection coefficients in the band of 5.75–5.84 GHz of the proposed array antenna are below $10$ dB in all scanning status. The radiation efficiency of the proposed array antenna shows more than 75% in the operating bandwidth of all switching modes.

The simulated scanning performance of the proposed array antenna in xz-plane at 5.8 GHz is shown in Fig. 11. The scanning patterns shown in Fig. 11 are the simulated results when the phase difference varies from ${-180}^{\circ }$ to ${180}^{\circ }$ with ${22.5}^{\circ }$ step, and only the cases when the maximum sidelobe level is less than $-10$ dB are shown in the figure. Figure 11(a–c) show the scanning patterns in Mode 1, Mode 2, and Mode 3, respectively, and it can be seen that only the range of Subspace I, Subspace II, and Subspace III is scanned in each mode. The scanning range of Subspace I, Subspace II, and Subspace III were ${-70}^{\circ }\sim {-10}^{\circ }$, ${-18}^{\circ }\sim 1{8}^{\circ }$, and ${10}^{\circ }\sim {70}^{\circ }$, respectively. The simulated total scanning range of the proposed array antenna was ${-70}^{\circ }\sim {70}^{\circ }$ and the peak gain was 17.6 dBi.

From the simulated results, it can be seen that there is no problem with the scanning performance even if the antenna elements are arranged with the spacing of 0.9${\lambda }_{0}$ because the active element patterns meet the guideline. To validate it experimentally, a prototype array antenna was fabricated. The photographs of the prototype array antenna are shown in Fig. 12. Figure 12(a) shows the whole structure of the beamforming system including jigs. Plastic jig, spherical dielectrics, and microstrip patches are fixed in place. After fixing the array antenna with plastic jig and acrylic plate, the beamforming network, control board and switching network were connected to the back of the acrylic plate using connectors. Figure 12(b) shows the beamforming network. The upper region of the figure is connected only to Port 1 of each antenna element, and the lower region is connected only to Port 2 of each antenna element. They can be selected alone or be selected simultaneously by using the switching network. Figure 12(c) shows the beamforming system configuration. The array antenna is connected to the beamforming network via the redistribution layer, the switching network selects the switching mode, and the beamforming network is controlled by the control board. All of these boards can be easily connected and disconnected using connectors. Figure 12(d,e) show the photographs of the assembled beamforming system and the measurement environment, respectively.

The measured scanning performance of the prototype array antenna in xz-plane at 5.8 GHz using anechoic chamber is shown in Fig. 13. Figure 13(a--c) show the measured scanning patterns in Mode 1, Mode 2, and Mode 3, respectively. The measured scanning range of Subspace I, Subspace II, and Subspace III were ${-70}^{\circ } \sim {-13}^{\circ }$, ${-17}^{\circ }\sim {16}^{\circ }$, and ${12}^{\circ }\sim {70}^{\circ }$, respectively. The measured total scanning range of the prototype array antenna was ${-70}^{\circ }\sim {70}^{\circ }$ and the peak gain of the system was 22.9 dBi. Considering the LNA gain (16.1 dB), phase shifter loss ($-6$ dB), total line loss ($-1.2$ dB), connector loss ($-1$ dB), and switching loss ($-1.96$ dB), the peak gain of the prototype array antenna can be estimated about 17 dBi. The measured results showed reasonable agreement with the simulated results and the wide-angle scanning performance of the proposed array antenna was verified.

## Discussion

In this paper, we presented the design, fabrication, and measurement of a wide-angle scanning phased array antenna using high gain PRA elements. Because the array pattern of phased array antenna is the product of array factor and element pattern, if the antenna element can reconfigure its beam pattern, the array beam can be scanned by dividing the scanning range into several subspaces. In addition, this solution is not restricted by the element spacing because the grating lobes can be utilized as the main beam. To achieve the desired scanning range of the phased array antenna, some characteristics of the PRA element such as the number of switching modes, tilt angle in each mode, and maximum HPBW are required. We derived the required characteristics of PRA element depending on the target scanning range and element spacing. Based on the derivation, a PRA element design guideline for phased array antennas was presented. When the active element patterns of the PRA elements meet the guideline, the desired scanning performance can be achieved by dividing the scanning range into several subspaces.

In accordance with the guideline, the target scanning range was set as $\pm {70}^{\circ }$ and an 8x1 array antenna was composed using a high gain PRA model. The model is a high gain SDRA operating on higher-order resonant mode which can reconfigure its beam pattern to three directions. Considering the size and the number of switching modes, the elements were arranged with 0.9${\lambda }_{0}$ spacing and analyzed whether the active element patterns have suitable characteristics. The tilt angles and HPBWs of the active element patterns showed suitable characteristics to the desired scanning performance. Thereafter, an 8x1 phased array antenna composed of the high gain PRA elements was proposed. The simulated results showed that the proposed array antenna can scan the desired scanning range by dividing the range into three subspaces. To validate the scanning performance experimentally, a prototype array antenna was fabricated and measured. The measured peak gain of the prototype array antenna was estimated about 17 dBi and the measured scanning range was from ${-70}^{\circ }$ to ${70}^{\circ }$ with the gain fluctuation less than 3 dB. In all scanning range, the side lobe level was less than $-10$ dB. The measured results showed good wide-angle scanning performance which validates the effectiveness of the proposed method. Since the antenna element is pattern reconfigurable and has high gain, a wide-angle scanning performance with high array gain can be obtained with a small number of antenna elements.

The proposed wide-angle scanning technique has many advantages but there are some caveats. Firstly, since the switches are added to consist the switching network for pattern reconfiguration, the complexity and loss of the system are increased. To compensate for this drawback, it is necessary to increase the antenna element gain by applying various techniques for gain enhancement, such as using higher-order resonant mode, as in this paper. The proposed wide-angle scanning technique has advantages by using a high gain PRA element that can sufficiently compensate for the increased complexity and loss of the system. Secondly, when practically operating the phased array antenna composed of PRA elements, the complexity of the beamforming method increases because not only the phase difference but also the switching mode of the PRA must be considered. In addition, since the beamforming is performed using not only the main lobe of the array factor but also the grating lobes, it becomes more complicated to control the phase shifter than the beamforming method only using the main lobe. When mode switching occurs, the phase difference should be properly adjusted for continuous beam scanning. To ensure the smooth beam scanning, more accurate beam control is required based on the sufficient simulation and measurement data. With these precautions in mind, for the desired scanning performance, it is important to design the optimal antenna according to the guideline and to accurately control the beam with consideration of the switching mode.

## Methods

The proposed array antenna and PRA element were designed and simulated using CST Microwave Studio. The operating frequency of the element and array was set as 5.8 GHz, which is the ISM band. The beamforming network, switching network, and control board were designed and simulated using Keysight Advanced Design System. In Fig. 12, eight dielectric spheres were fabricated by ceramic material (${\varepsilon }_{r}=13$, $tan\delta =0.02$). The microstrip patches were fabricated using 0.76 mm thickness substrate Taconic RF-301 (${\varepsilon }_{r}=2.97$, $tan\delta =0.0012$). The beamforming network and control board were fabricated using 0.254 mm thickness substrate Taconic RF-35 (${\varepsilon }_{r}=3.5$, $tan\delta =0.0018$). The reconfigurable switching network was fabricated using 0.254 mm thickness substrate Taconic TLY-5A (${\varepsilon }_{r}=2.2$, $tan\delta =0.0009$). The redistribution layer for matching the delay between the beamforming network and each port of antenna elements was fabricated using 0.254 mm thickness substrate Taconic RF-30 (${\varepsilon }_{r}=3.0$, $tan\delta =0.0014$). The plastic jig was fabricated using 3-D printer and polylactic acid (${\varepsilon }_{r}=3.5$, $tan\delta =0.001$). The jig fixes the dielectric spheres above the microstrip patches. The far-field radiation patterns of the prototype array antenna in Fig. 13 were measured in the anechoic chamber of Daeduk Radio Engineering Center. The scanning performances of each mode were obtained by measuring the far-field radiation patterns of the array antenna while varying the phase difference.
